# Serum and tissue expression of neuropilin 1 in precancerous and malignant vocal fold lesions

**DOI:** 10.1371/journal.pone.0239550

**Published:** 2020-10-01

**Authors:** Anna Rzepakowska, Michał Żurek, Jakub Grzybowski, Iwona Kotula, Paweł Pihowicz, Barbara Górnicka, Urszula Demkow, Kazimierz Niemczyk

**Affiliations:** 1 Department of Otorhinolaryngology Head and Neck Surgery, Medical University of Warsaw, Warsaw, Poland; 2 Students Scientific Research Group at the Department of Otorhinolaryngology Head and Neck Surgery, Medical University of Warsaw, Warsaw, Poland; 3 Department of Pathology, Medical University of Warsaw, Warsaw, Poland; 4 Department of Laboratory Diagnostics and Clinical Immunology of Developmental Age, Medical University of Warsaw, Warsaw, Poland; University Hospital Eriangen at Friedrich-Alexander-University Erlangen-Numberg, GERMANY

## Abstract

**Objectives:**

The study was designed to evaluate the tissue expression of NRP-1 and serum level of sNRP-1 in the same patients with intraepithelial laryngeal lesions or early staged laryngeal cancer to identify the clinical significance of these biomarkers in the diagnosis of laryngeal lesions.

**Material and methods:**

A prospective analysis of tissue was performed on specimens and blood samples from 49 patients, who were admitted for surgical resection due to suspicious vocal fold lesions and were diagnosed as non-dysplasia, low-grade dysplasia, high-grade dysplasia and invasive cancers.

**Results:**

ELISA was conducted on 48 blood samples. The minimum level of sNRP-1 was 0.15 ng/ml and maximum– 37.71 ng/ml. The Kruskal–Wallis one-way analysis of variance revealed no differences in sNRP-1 levels between different histopathological stages of vocal fold lesions (p = 0.234). IHC was conducted in 49 tissue samples. The evaluated mean scores of NRP-1 tissue expression were compared to histopathological stage of the lesion. The Kruskal–Wallis one-way analysis of variance revealed no differences in NRP-1 tissue expression between different histopathological stages of vocal fold lesions (p = 0.536). The correlation of tissue NRP-1 expression and serum levels of NRP-1 within analyzed group was insignificant. The Spearman’s rank correlation coefficient was 0.076 (p = 0.606).

**Conclusions:**

The NRP-1 tissue expression and serum levels are unlikely to be a prognostic factor for identification of laryngeal dysplasia or early stage laryngeal cancer. Further studies investigating biomolecules involved in laryngeal carcinogenesis are necessary.

## Introduction

The larynx remains the most common site of head and neck squamous cell carcinoma. According to the estimation of International Agency for Research on Cancer the number of new cases in 2018 reached 177 422 worldwide, and the prognosis for 2040 predicts increase to 285 720 [1]. Tobacco and alcohol consumption are confirmed risk factors predisposing to development of squamous cell carcinoma within the larynx [2–4]. The role of laryngopharyngeal reflux in the laryngeal cancer initiation is still controversial [5, 6].

The majority of laryngeal cancer cases develop on a background of precancerous lesions of the mucosa [3, 4, 7]. The hypertrophic lesions of the laryngeal mucosa are common pathologies in clinical practice and only a few are associated with the development of invasive cancer. They represent inflammatory, non-dysplastic, hyperkeratotic, dysplastic, preinvasive cancer and early invasive cancer. This wide range of histopathological disease stages present clinically with identical symptoms and the similar appearance in white light examination. The novel endoscopic method of narrow band imaging increased the accuracy of clinical differentiation of high grade dysplasitic lesions and early cancer from non-dysplastic pathologies [8]. Although the method has high sensitivity and specificity, false negative or false positive cases may occur. Presently, the biopsy under the direct laryngoscopy is still the standard diagnostic procedure for final diagnosis. Identification of specific biomarkers of laryngeal intraepithelial dysplasia and/or cancer transformation could improve the accuracy of clinical investigation.

Several authors have made the attempt of searching for markers expressed in tissue specimens and/ or in the blood in patients with laryngeal cancer, however no marker has demonstrated a high enough specificity [9–11]. Angiogenesis seems to be an essential process in the progression of non-dysplastic laryngeal lesions into the dysplasia [12]. The predictive value of angiogenic factors in laryngeal cancer is the subject of contemporary researches with the majority concentrating on the activity of vascular endothelial growth factor (VEGF) and vascular endothelial growth factor receptor (VEGFR) axis regulated by transcription factors such as hypoxia inducible factor 1α (HIF-1α) and co-receptors such as vascular endothelial growth factor receptor 1 (VEGFR1) and Neuropilins 1 and 2 (NRP 1, 2). Recent studies confirmed the negative correlation of disease free survival with high expression of vascular endothelial growth factor (VEGF) and CD31 in early glottic cancer [13, 14]. Pentheroudakis et al. analyzed mRNA extracted from 229 laryngeal cancer patients and revealed high VEGFR1 mRNA level within the tumour associated with high risk for recurrence and decreased progression-free and overall survival [15].

Novel oncological therapies target VEGF and its receptors VEGFR-1, VEGFR-2 and NRP-1. NRP-1 is a transmembrane glycoprotein with a large extracellular and short cytoplasmatic domain. It is a coreceptor for some growth factors, including transforming growth factor-β (TGF- β), platelet-derived growth factor (PDGF) and primarily VEGF [16–19]. Each cell of the vascular system expresses NRP-1, because of its crucial role in developing and maturing of blood vessels [16, 18, 19]. Upregulation of NRP-1 was reported in several cancers: oral squamous cell carcinoma, cervix cancer, hepatocellular cancer [17–20]. There are sill very limited data concerning the role of NRP in progression of laryngeal cancer. The only available study of Pentheroudakis et al. did not revealed prognostic impact of Neuropilins 1 and 2 mRNA expression on the outcomes [15]. Neuropilin-1 can be also detected as a soluble protein (sNRP-1). sNRP-1 acts as a competitive antagonist of growth factors and is a natural inhibitor of angiogenesis [18, 19]. There are studies confirming sNRP-1 as potential biomarker in breast and cervix cancer [20, 21].

In this study the tissue expression levels of NRP-1 and serum level of sNRP-1 in the same patients with intraepithelial laryngeal lesions or early staged laryngeal cancer were evaluated and compared to identify the clinical significance of these biomarkers in the diagnosis of laryngeal lesions.

## Material and methods

The protocol of the study was approved by the Ethics Committee at the Medical University of Warsaw. Informed written consents were obtained from all patients. All procedures performed in the study were in accordance with the ethical standards of the national research committee and with the 1964 Helsinki declaration and its later amendments.

Participants were randomly recruited from patients hospitalized and treated in the Department of Otorhinolaryngology, Head and Neck Surgery in time periods: from January 2018 to March 2018 and from September 2018 to December 2018.

A prospective analysis of tissue was performed on specimens and blood samples from 49 patients, who were clinically diagnosed with suspicious laryngeal lesions of hypertrophy, leukoplakia and/or erythroplakia and were admitted to the otolaryngology department for surgical resection. We excluded patients, who had a history of systemic immunotherapy, chemotherapy, diagnosed neoplasms in other locations, or positive serological tests for hepatitis B and/or C. Lin et al. demonstrated increased level of sNRP-1 in patients with the hepatitis B, hepatitis C and cirrhosis comparing to heathy controls [21].

Transoral laryngeal microsurgery was performed in each patient and the lesion was resected with the CO_2_ laser. The final diagnosis was confirmed on histopathological examination according to World Health Organization (WHO) classification of laryngeal precancerous lesions from 2017, which includes four grades: non-dysplasia, low-grade dysplasia, high-grade dysplasia (optionally in severe cellular changes carcinoma in situ may be separated within this group) and invasive cancers [22].

Formalin-fixed and paraffin embedded laryngeal specimens were stored in a biological resources repository of the pathomorphology department. The peripheral blood samples, that were collected in the morning before surgical excision of the laryngeal lesion, and then centrifuged at 4ºC to retrieve the serum, were stored at –80°C in the biological resources repository at the department of laboratory diagnostics. National ethical guidelines of human tissues collecting were preserved.

### Immunohistochemistry (IHC)

**Fig 1:**
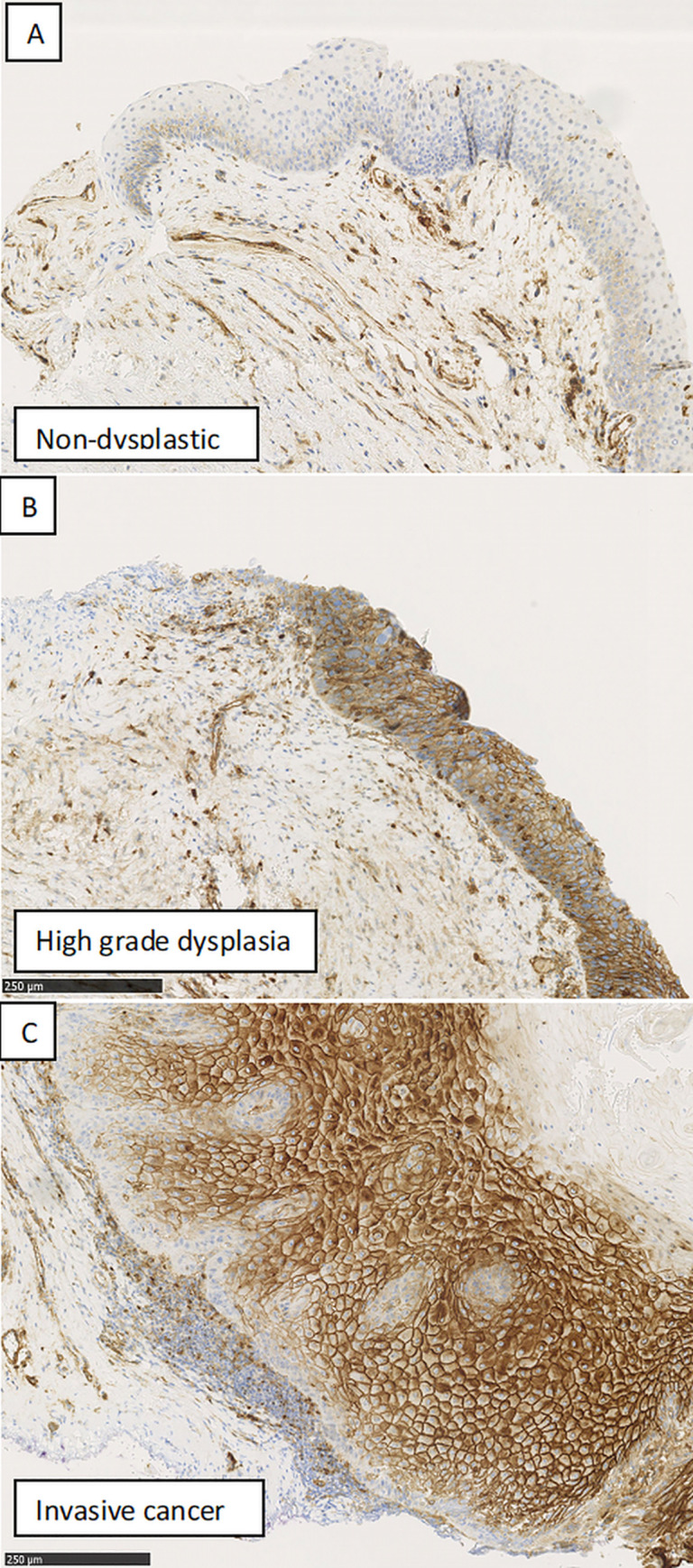
The categories of the expression of NRP-1 immunostaining in the intraepithelial vocal fold lesions at x100 magnification: A. weakly positive expression, B. moderately positive expression, C. strong expression.

The laryngeal specimens were deparaffinised and incubated with EnVision™ FLEX Peroxidase-Blocking Reagent (RTU) to block activity of endogenous peroxidase. Primary antibodies against NRP-1 (Abcam, Cambridge, UK, ab81321) were applied based on their specificity, followed by treatment with EnVision™ FLEX /HRP (RTU) and Envision™ FLEX Substrate Working Solution as a visualization system. All immunostained tissue sections were then counterstained with hematoxylin (EnVision™ FLEX Hematoxylin (RTU)). All steps were separated by using Rinse Wash Buffer. Tissue sections were dehydrated using a series of alcohol solutions. In the end, samples were closed with Mounting Medium Pertex® Histolab and coverslips. All stained sections were imaged with a microscope and 4 high-power visual fields with 500 atypical or normal laryngeal cells were selected and evaluated independently by two pathologists (J.G. and P.P.) The obtained results were averaged. The expression of NRP-1 in laryngeal lesions was evaluated semi-quantitatively. The percentage of positively stained cells (p) for a given intensity of staining (i) was determined by adding and averaging the result for each tested section. The intensity (i) of staining was assessed with following scores: 1—weak or no staining, 2—moderate staining, 3—strong staining. The final score (R) was obtained by multiplying the percentage of positive cells (p) (1–100%) by the intensity (i). The formula was R = p×i/100, range: minimum = 1, maximum = 3. (Fig 1).

### Enzyme-linked immunosorbent assay (ELISA kit)

Peripheral serum samples were aliquoted and frozen to a temperature of –80°C. The blood samples were thawed shortly before determination of biomarker expression by ELISA assay. A sandwich enzyme immunoassay using monoclonal anti-NRP-1 antibodies (Shanghai Sunredbio Technology Co.,Ltd, Catalogue No. 201-12-4380) and enzyme-linked polyclonal antibodies was performed. The ELISA procedures were performed according to the manufacturer’s instruction. Individual serum concentration of the protein was reported in μg/ml.

### Statistical analysis

Serum and tissue expression of NRP-1 were tested for correlation with the patient’s histopathological disease stage, age and sex using Mann–Whitney U test and Spearman's rank correlation coefficient. Kruskal–Wallis one-way analysis of variance was used to check the differences in NRP-1 values between groups. Correlation between sNRP-1 and tissue expression of NRP-1 was analyzed by Spearman's rank correlation coefficient. Analysis was performed using IBM SPSS Statistics 25.0. A minimum p< 0.05 was considered to be statistically significant.

## Results

49 patients (35 men, 16 women; mean age 63.59 years, SD 12.93) with vocal fold lesions were included into the study. The final histopathological diagnosis of the lesions included 25 non-dysplastic lesions, 6 low-grade dysplasia, 10 high-grade dysplasia and 10 invasive cancers of T1 stage. Demographical data of the patients are presented in Table 1.

**Table 1:** Demographical data of the study group.

Data	Case No (%)
Female	16 (32.7%)
Male	33 (67.3%)
Mean age /median/SD (years)	63.45 /62/ ±12.47
Smokers (active or abstinence shorter than 6 months)	40 (81.6%)
Histopathological diagnoses (%)	
Non-dysplastic lesions	22 (44.9)
Low-grade dysplasia	8 (16.3)
High-grade dysplasia	9 (18.4)
Invasive cancer	10 (20.4)

### NRP-1 serum expression (sNRP-1) with ELISA test

ELISA was conducted on 48 blood samples. Sensitivity of the test was 0.109 ng/ml and the linear range was from 0.15 to 40 ng/ml. The minimum level of sNRP-1 was 0.15 ng/ml and maximum– 37.71 ng/ml. The values of sNRP-1 levels according to the histopathological diagnosis and sex are presented in Table 2.

**Table 2:** The values of NRP-1 serum expression (sNRP-1) according to the histopathological diagnosis and sex (ELISA test).

	Mean value (ng/ml)	No of samples	Standard deviation (ng/ml)	Minimum (ng/ml)	Maximum (ng/ml)	p-value
Histopathological diagnosis						
Non-dysplastic lesions	6.3	22	9.71	0.15	29.92	0.234
Low grade dysplasia	7.74	7	10.47	0.15	30.55	
High grade dysplasia	14.57	9	15.56	0.15	37.71	
Invasive cancers	2.32	10	3.28	0.16	8.30	
Sex						
Women	12.94	16	13.93	0.15	37.71	**0.029**
Men	4.76	32	8.13	0.15	30.23	

p< 0.05 was considered to be statistically significant

The Kruskal–Wallis one-way analysis of variance revealed no differences in sNRP-1 levels between different histopathological stages of vocal fold lesions (p = 0.234).

**Fig 2:**
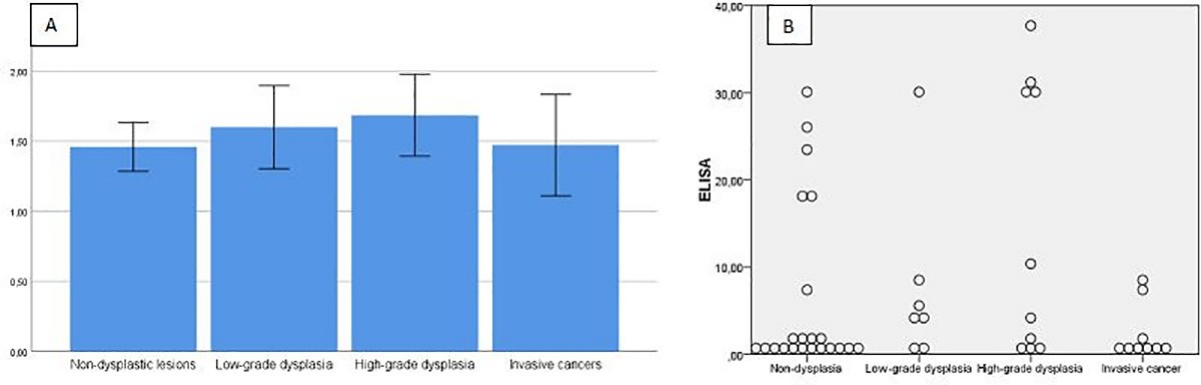
Graphical presentations of Elisa results in the study group with the bar graph of mean sNRP-1 levels including error bars (A) and with the 2-D dot plot (B).

Fig 2 graphically presents sNRP-1concentrations of Elisa results in the study group with the bar graph of mean sNRP-1 levels including error bars (A) and with the 2-D dot plot (B) (Fig 2).

The Mann–Whitney U test confirmed a significant difference in sNRP-1 levels between female and male patients (p = 0.029). The correlation of patients age with the levels of sNRP-1 was insignificant. The Spearman’s rank correlation coefficient was 0.06 (p = 0,686).

### NRP-1 tissue expression by immunohistochemistry

IHC was conducted in 49 tissue samples of vocal fold lesions. The results of evaluated mean scores of NRP-1 tissue expression according to the histopathological stage of the lesion and patients sex are presented in Table 3.

**Table 3:** The mean scores of immunohistochemistry NRP-1 tissue expression according to the histopathological stage of the lesion and patients sex.

	Mean Score	No of tissue samples	Standard deviation	Minimum	Maximum	p-value
Histopathological diagnosis						
Non-dysplastic lesions	1.459	22	0.390	1.00	2.00	0.536
Low grade dysplasia	1.600	8	0.354	1.10	2.00	
High grade dysplasia	1.685	9	0.408	1.10	2.50	
Invasive cancers	1.472	10	0.469	1.00	2.20	
Sex						
Women	1.546	16	0.363	1.00	2.00	0.797
Men	1.522	33	0.425	1.00	2.50	

p< 0.05 was considered to be statistically significant

**Fig 3:**
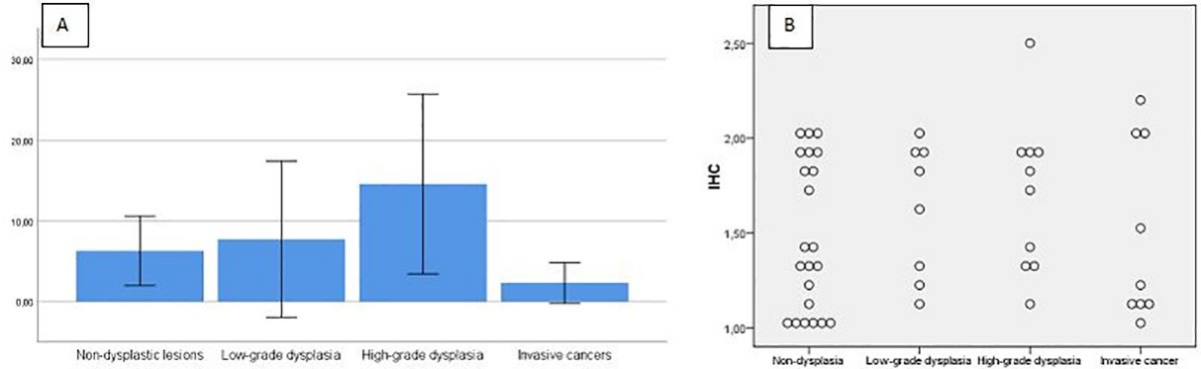
Graphical presentations of immunohistochemistry results of NRP-1 tissue expression in the vocal folds lesions with a bar graph of mean scores including error bars (A) and with a 2-D dot plot (B).

The Kruskal–Wallis one-way analysis of variance revealed no differences in NRP-1 tissue expression between different histopathological stages of vocal fold lesions (p = 0.536). Fig 3 graphically presents immunohistochemistry results of NRP-1 tissue expression in the vocal folds lesions with a bar graph of mean scores of NRP-1 including error bars (A) and with a 2-D dot plot (B) (Fig 3).

The Mann–Whitney U test revealed no difference in NRP-1 tissue expression between female and male patients (p = 0.797). The correlation of patients age with the tissue expression of NRP-1 was insignificant. The Spearman’s rank correlation coefficient was 0.154 (p = 0.292).

The correlation of tissue NRP-1 expression and serum levels of NRP-1 within analyzed group was insignificant. The Spearman’s rank correlation coefficient was 0.076 (p = 0.606).

## Discussion

Clinicopathological features of laryngeal cancer such as stage, grade, nodal status are confirmed predictors of recurrence, metastasis and overall survival. However head and neck surgeons still search for reliable biomarkers associated with histopathological features and that correspond to biological behavior of the disease. Credible markers are needed that could be applied in the screening of risk groups, efficiently differentiating between precancerous and invasive conditions as well as capable of detecting the recurrence or metastases even before there is evidence in clinical or radiological imaging methods. In the present era with the growing importance of personalized therapy in oncology, such biomarkers may also provide potential targets for therapy with constructed antibodies.

The role of angiogenesis in solid tumour growth is essential and the most studied angiogenic cytokine is vascular endothelial growth factor (VEGF) produced by tumour and stromal cells. The results of meta-analysis that included 12 studies comprising 1002 patients showed that overexpression of VEGF receptor (VEGFR) in 90% of head and neck squamous cell carcinomas (SCC) and the up-regulation of VEGFR was associated with poorer survival outcomes [23]. However the results of currently performed trials analyzing the addition of bevacizumab, a monoclonal antibody targeting VEGF, to platinum-based chemotherapy in recurrent or metastatic SCC of the head and neck, did not demonstrate efficacy in increasing overall survival but improved the response rate and progression-free survival [24]. These results encourage further biomarker-driven studies to identify other more effective angiogenesis inhibitors. The activity of the VEGFR depends not only on VEGF concentration, but also on co-receptors such as VEGFR1 and Neuropilins 1 and 2. The expression of NRP1 was analyzed in different solid carcinomas including bladder, breast, colon, lung, ovarian and prostate and the up regulation of the biomolecule was correlated with advanced tumor stage and/or disease progression [17–20]. A study by Mehta et al. identified correlation of high levels of NRP-1 in head and neck SCC samples with decreased survival and earlier progression of disease [25]. Also the results of Al-Shareef et al. suggests that NRP-1 expression is an independent factor that is likely to predict the risk of lymph node metastasis in SCC of the tongue [26].

In the present study we sought to evaluate the expression of NRP-1 during laryngeal intraepithelial lesions progression. The idea for this study was derived from the promising results of studies evaluating NRP-1 in head and neck SCC as well as the results of two other studies assessing NRP-1 levels in dysplastic and early invasive lesions. Yang et al. confirmed significant upregulation of circulating and tissue expressed NPR-1 in patients with cervical cancer and dysplastic intraepithelial lesions compared to the control group. The authors suggested that the process of NRP-1 upregulation is initiated at early stages of dysplasia formation and the increase of NRP-1 levels correlated with cervical cancer stage and nodal metastases [20]. Another study by Shahrabi-Farahani et al. revealed significant up-regulation of NRP-1 in oral epithelial dysplasia and oral squamous cell carcinoma (OSCC) and showed increased expression of NRP-1 in dysplastic tongue epithelium and OSCC not only within the basal but also throughout proliferating epithelial layers [17]. Our study was the first investigating NPR-1 expression in laryngeal intraepithelial lesions and early staged glottic cancer. We assumed the hypothesis that NRP-1 is up-regulated throughout the stages of laryngeal carcinogenesis and correlates with atypia progression and invasiveness of laryngeal SCC. However the preliminary results of our study did not detect any association between neither the tissue or serum expression level of NRP-1 with histopathological grade of laryngeal intraepithelial lesions. Our results are therefore in contrast with Yang et al. and Shahrabi-Farahani et al. studies, that evaluated IHC staining of NRP-1 in squamous cancer cell and its precancerous lesions of other sites. Such inconsistency between our and Yang et al. study results might emerge from their larger samples within subgroups and from the inclusion of the control group with healthy uterine cervical tissue samples into their analysis as well as the subgroup of advanced stage cancers. Most of their significant differences of sNRP-1 levels and tissue expression of NRP-1 were referred to healthy control group. In our study we assumed the non-dysplastic laryngeal lesions as potential control group for comparison with dysplasia and early invasive cancer. Considering that the hypoxic genetic pathways are commonly activated in hyperproliferative lesions and promote angiogenesis through HIF-1α and that NRP-1 expression is significantly increased in hypoxic tumor microenvironment [27], together with the results of Rzepakowska et al. study presenting immunohistochemical staining of laryngeal specimens for HIF-1α and confirming strong expression of the marker throughout all hypertrophic laryngeal lesions with 60% of stained cells in non-dysplastic lesions, 100% in low-grade dysplasia, 53% in high-grade dysplasia and 50% in invasive cancers [12], it would be recommended to verify the sNRP-1 and tissue NRP-1 expression in samples from patients without any pathological changes of vocal folds. The sampling of healthy vocal fold mucosa carries however a risk of scarring with permanent voice quality deterioration and is therefore avoided. The work by Shahrabi-Farahani et al. presented only images of individual cases of tongue normal tissue, dysplasia and cancer and revealed upregulated NRP-1 tissue expression however without any statistical comparison. Another solution for verifying the role of NRP-1/VEGF axis in the progression of laryngeal dysplasia and invasive cancer may be construction of the study with molecular assessment of NRP-1 and VEGFR gene transcription and comparing them with protein expression.

Yet another issue to raise is the technique of specimen collection. Currently the majority of laryngeal hypertrophic lesions are resected with the CO2 laser during the transoral microsurgery procedure. The advantages of laser comparing to cold instrument resection is the combined function of cutting and coagulation of the laser with magnified visualization of the surgical microscopes and precise direction of the laser beam with the micromanipulator. In our study all laryngeal samples were collected during the laser microsurgery and the whole lesion was thoroughly resected and fixed in formalin for histopathology. The differences in IHC staining of laryngeal specimens related to cold instruments or CO2 laser resection are raising questions. The thermal injury and coagulation of tissue margin is inevitable using laser beam. Although the IHC assessment of markers expression is made in well preserved central parts of the infiltration, the increased temperature spread wider on cells and may further influence their ability of binding antibodies. However we did not found reliable studies comparing the staining effectiveness with relation to the method of dissection.

The major limitation of our study is the small amount of analyzed groups. We also did not analyze comorbidities or the status of addictions in our patients, because a reliable biomarker should not be dependent on other variables.

## Conclusions

The NRP-1 tissue expression and serum levels are unlikely to be a prognostic factor for identification of laryngeal dysplasia or early stage laryngeal cancer. Further studies investigating biomolecules involved in laryngeal carcinogenesis are necessary.

## Supporting information

S1 File (XLSX)
